# Sex-specific association of body mass index and fatty liver index with prevalence of renal hyperfiltration: a cross sectional study using Japanese health check-up data

**DOI:** 10.1186/s12882-023-03137-x

**Published:** 2023-04-03

**Authors:** Atsushi Kitazawa, Yoshiharu Fukuda

**Affiliations:** 1 Teikyo University Graduate School of Public Health, 2-11-1 Kaga, Itabashi-Ku, Tokyo, Japan; 2 Department of Nephrology, Dokkyo Medical University Saitama Medical Center, Saitama, Japan

**Keywords:** Renal hyperfiltration, BMI, FLI, NAFLD, Obesity, BSA, CKD-EPI

## Abstract

**Background:**

The relationship between obesity and nonalcoholic fatty liver disease and renal hyperfiltration is controversial. This study aimed to assess the correlations of body mass index and fatty liver index, respectively, with renal hyperfiltration in non-diabetic subjects, considering age, sex, and body surface area.

**Methods:**

This cross-sectional study assessed the Japanese health check-up data (FY2018) of 62,379 non-diabetic individuals from a health insurance database. Renal hyperfiltration is the ≥ 95th percentile of estimated glomerular filtration rate (derived by Chronic Kidney Disease Epidemiology Collaboration formula) by gender and age in healthy subjects. After adjusting for potential confounders, multiple logistic regression models were applied to evaluate the correlation of renal hyperfiltration with body mass index categories and fatty liver index (10 equal parts).

**Results:**

A negative and positive correlation, respectively, were noted when the body mass index was < 21 and ≥ 30 in women; however, a positive correlation was noted for BMI < 18.5 and ≥ 30 in men. Renal hyperfiltration prevalence increased when fatty liver index increased for both sexes; the cutoff value for fatty liver index was 14.7 for women and 30.4 for men.

**Conclusions:**

Body mass index and renal hyperfiltration correlated linearly in women; however, in men, the correlation was U-shaped; therefore, differing by sex. However, fatty liver index correlated linearly with renal hyperfiltration in both sexes. Non-alcoholic fatty liver disease might be associated with renal hyperfiltration; Fatty liver index is a simple marker that can be obtained from health check-ups. Since a high fatty liver index correlated with renal hyperfiltration, it may be beneficial to monitor the renal function in such a population.

## Background

Renal hyperfiltration (RHF) is a well-known phenomenon that occurs early in the development of nephropathy in patients with diabetes [1]. RHF is thought to be followed by the development of proteinuria and progressive decline in renal function [2]. Recently, it has been reported that RHF correlates with prediabetes [3–6], making it an early marker for the onset of diabetes. RHF is associated with renal prognosis and all-cause mortality in patients with diabetes [7].

The following points are important when investigating RHF: 1) the definition of RHF; 2) the method of measurement of glomerular filtration rate (GFR); and 3) whether GFR should be adjusted for body surface area (BSA).

1) There is no universal definition of RHF. Some studies have defined RHF as estimated glomerular filtration rate (eGFR) ≥ 120 ml/min [8], while others have used eGFR to define RHF as the 95th percentile or + 2 SD in healthy subjects. According to a systematic review by Cachat et al., 30% of the studies did not justify the choice of the threshold values [9]. From a methodological point of view, they argued that an age-and gender-matched control group should be used to define the RHF threshold.

2) The gold standard for GFR measurement is the inulin clearance test; however, it is not performed in epidemiological studies because it is a complex and time-consuming test. In clinical practice, eGFR, which is estimated from serum creatinine (Cr) values, is used as a measure of GFR. Different formulas are used to determine eGFR across regions and countries; notably, the serum Cr levels vary according to sex, age, race, and other factors. The Modification of Diet in Renal Disease Study (MDRD)-eGFR and Chronic Kidney Disease Epidemiology Collaboration (CKD-EPI)-eGFR formulas are the most commonly used formulas for estimating the GFR. In Japan, the MDRD-eGFR formula has been modified for use in the Japanese population and the modified formula is widely used [10]. For men, eGFR (ml/min/1.73 m^2^) = (194 × Cr -1.094 × Age-0.287), and for women, it was further multiplied by 0.739. However, the MDRD-eGFR equation was developed mainly for chronic kidney disease (CKD) patients; therefore, when it was applied to patients with normal renal function (eGFR ≥ 60), the GFR was estimated to be low in several cases [11]. The CKD-EPI equation was developed to improve on this point by using different equations for estimating the eGFR according to the serum Cr levels (Cr 0.9 for men and 0.7 for women) [12]. The coefficient in the CKD-EPI formula modified for the Japanese population was 0.813 [13]. The CKD-EPI equation has been noted to be a superior surrogate marker of GFR in patients with hyperfiltration [14]; additionally, the majority of studies on RHF based on eGFR used the CKD-EPI formula. However, studies on Japanese subjects are limited [4–6], and all of the studies used the MDRD-eGFR formula.

3) There is a clinicopathological syndrome associated with obesity called obesity-related glomerulopathy (ORG). The histological feature is glomerulomegaly, which may be due to increased metabolic demand, and functionally, there is an increase in the total glomerular filtration rate [15]. ORG has also been postulated to be a kidney lesion caused by metabolic syndrome. However, studies evaluating the relationship between obesity and RHF are controversial because the results vary depending on whether the GFR is indexed with BSA. Most previous RHF studies that evaluated GFR have found a positive relationship between the BMI and RHF that disappears upon adjustment of GFR to BSA [16–18]. The indexed GFR with BSA in obese individuals may underestimate the GFR. There are few large cohort studies of RHF using estimated GFR that have evaluated its correlation with BMI.

In addition to RHF, non-alcoholic fatty liver disease (NAFLD) is an independent risk factor for cardiovascular diseases. Recently, apart from the general cardiorenal risk factors, such as obesity, hypertension, diabetes, and hyperlipidemia, a strong association between the presence and severity of NAFLD and the prevalence and incidence of CKD has been clarified [19]. It has been suggested that insulin resistance may be a common pathogenic mechanism in NAFLD and CKD [20]. However, only one study has indicated an association between NAFLD (diagnosed by ultrasound or MRI) and RHF [21]. In that study, eGFR was converted to absolute value (mL/min) using the following equation: (eGFR mL/min/1.73 m^2^ * BSA)/1.73 m^2^. BSA was calculated using the DuBois and DuBois formula (BSA = 0.007184 × Weight^0.425^ × Height^0.725^) [22]. Patients with NAFLD presented higher levels of eGFR and a significantly increased prevalence of hyperfiltration (73.2%) compared to the patients without NAFLD. Moreover, NAFLD and increased weight were associated with an increased probability of hyperfiltration.

The diagnosis of NAFLD is usually made by ultrasonography; however, as a simpler marker, the fatty liver index (FLI), which can be calculated from the BMI, waist circumference (WC), triglyceride (TG), and gamma-glutamyl transferase (GGT) was reported by Bedgni et al. [23], and validation studies have been carried out in each region since it was first reported. In addition, there have been several studies showing that FLI is not only a marker for NAFLD, but also a predictive marker for diabetes and CKD. However, the relationship between FLI and RHF has so far been reported in only one small cohort study in Finnish men [24]. In that study, no correlation was noted between the FLI and RHF; both were independently associated with all-cause and cardiovascular mortality.

The aim of the present study was to assess the correlations of BMI and FLI with RHF in non-diabetic subjects, taking into account the age, sex, and BSA. For the purpose of this study, RHF was defined as the 95th percentile or higher of CKD-EPI eGFR by sex and age in healthy subjects at health check-ups. In addition, the analysis was also adjusted for BSA.

## Methods

### Study design and data source

The present study was a cross-sectional study performed using the Japanese health check-up data pertaining to FY2018. The data were obtained from a health insurance association and comprised annual health check-up data collected from all prefectures in Japan other than Tokyo.

### Study subjects

**Fig. 1:**
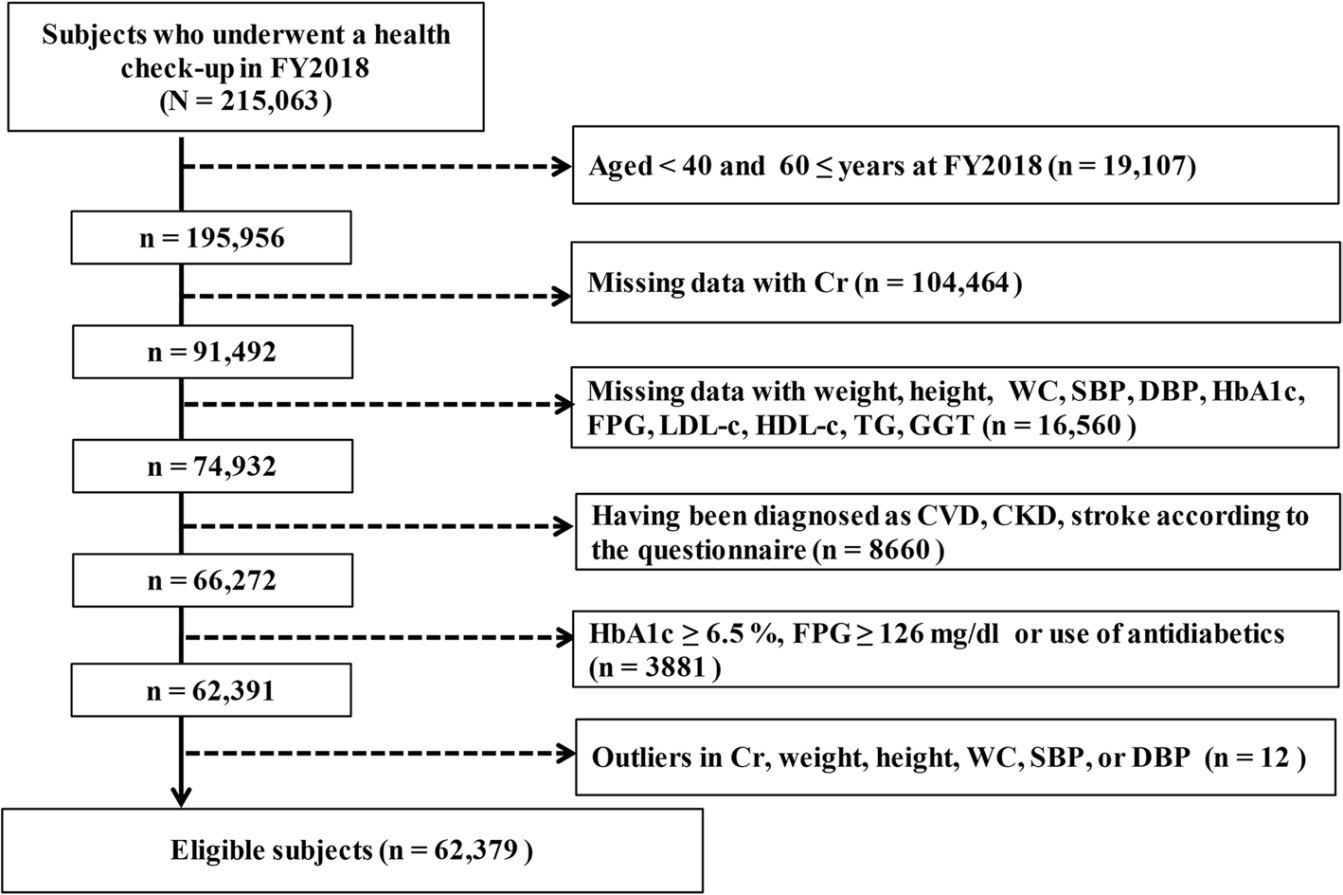
Flow of eligible subjects

The subjects were those aged 40–59 years who underwent a specific health check-up between April 2018 and March 2019. The eligible subjects for this study were those who (1) had all relevant data related to Cr, HbA1c (based on National Glycohemoglobin Standardization Program units), fasting plasma glucose (FPG), high-density lipoprotein cholesterol (HDL-c), low-density lipoprotein cholesterol (LDL-c), TG, GGT, systolic blood pressure (SBP), diastolic blood pressure (DBP), weight, height, and WC; (2) had no cardiovascular disease, chronic kidney disease, or stroke according to the questionnaire of the health check-up at FY2018; (3) did not have diabetes (HbA1c ≥ 6.5% or FPG ≥ 126 mg/dL or use of antidiabetics) in FY2018; and (4) had no outlier data for Cr, HbA1c, FPG, LDL, HDL, TG, GGT, weight, height, WC, SBP, or DBP at FY2018. The subjects who met all eligibility criteria are shown in Fig. 1.

### Definitions of renal hyperfiltration and hypofiltration

**Fig. 2:**
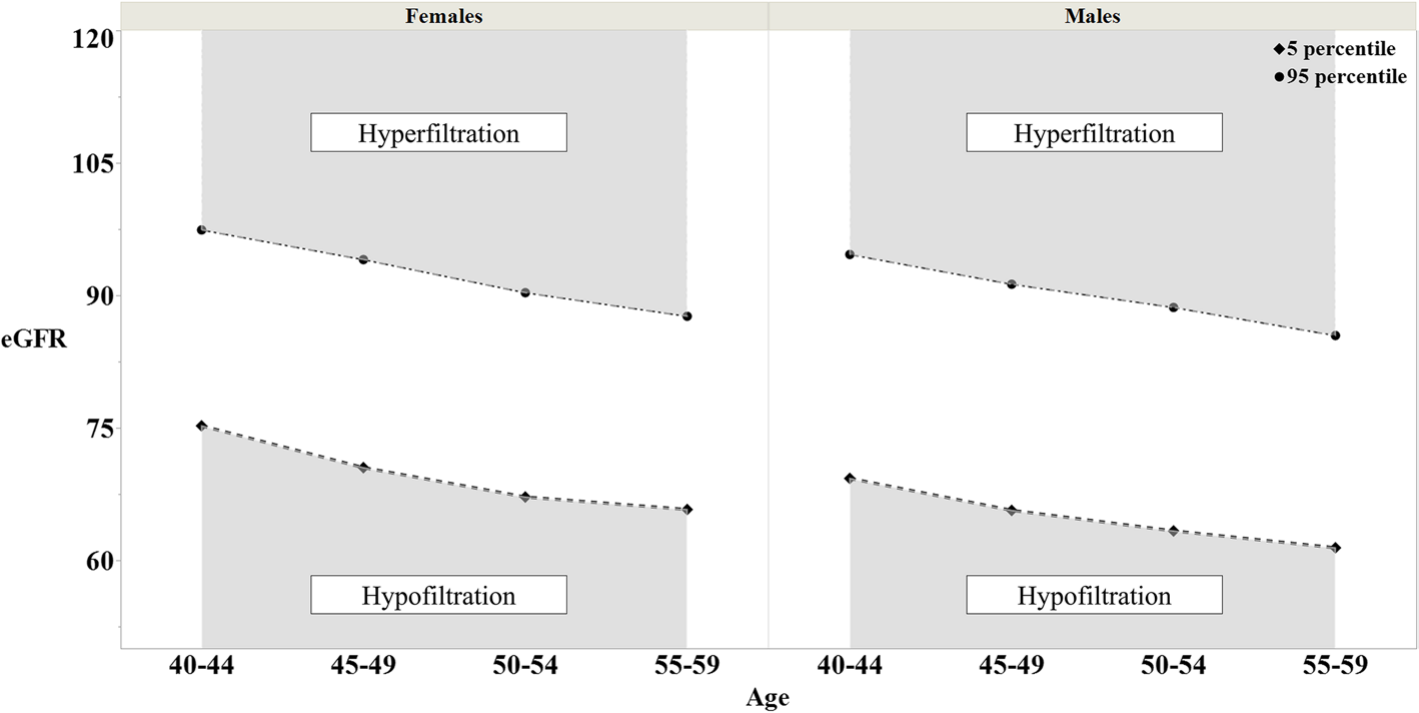
Distribution of eGFR (CKD-EPI) in "healthy subjects". The 95th and 5th percentiles are shown in 5-year age groups. Hyperfiltration was defined as an eGFR over the age-and sex-specific 95th percentile and hypofiltration was defined as an eGFR below the 5th percentile

The serum Cr levels were measured using enzymatic methods. The GFR was estimated from serum Cr values using the CKD-EPI formula [12] and adjusted using the Japanese coefficient, 0.813 [13]. The value of eGFR derived using this formula was indexed by BSA using the DuBois and DuBois formula [22]. To define hyperfiltration and hypofiltration, we focused on “healthy subjects” who met the following criteria: (1) No medication for hypertension, hyperlipidemia, or diabetes mellitus; (2) FPG < 100 mg/dL, HbA1c < 5.7%, SBP < 120 mmHg, DBP < 80 mmHg, LDL < 140 mg/dl, HDL ≥ 40 mg/dl, and TG < 150 mg/dl; and (3) a negative urine protein test. The “healthy subjects” were stratified into 8 groups according to sex and age (40–44, 45–49, 50–54, and 55–59 years), and hypofiltration and hyperfiltration were defined as values below the 5th percentile and above the 95th percentile of eGFR in each group, respectively. In addition, using the reference values, all subjects were divided into hypofiltration, normal filtration, and hyperfiltration groups based on their individual eGFR values. Subsequently, the subjects of the RHF were then compared with the subjects of normal filtration. A graph of the reference values for renal hyperfiltration and hypofiltration in women and men is shown in Fig. 2.

### Variables

For background variables, age, BMI, FLI, FPG, SBP, DBP, HDL-c, LDL-c, TG, and self-administered questionnaire (antihypertensive medication use, lipid-lowering medication use, current smoking, daily drinking, exercising for 30 min or more per day, skipping breakfast, midnight eating, weight gain of 10 kg or more since 20 years of age, and adequate sleeping) at FY2018 were extracted from the database. The BMI was calculated as the weight divided by height in square meters, and the FLI score was calculated as follows:

$$FLI= \left\{\frac{{e}^{0.953 \times \mathrm{log }\left(TG\right) + 0.139 \times BMI + 0.718 \times \mathrm{log }\left(\gamma - GTP\right) + 0.053 \times WC - 15.745}}{1+({e}^{0.953 \times \mathrm{log }\left(TG\right) + 0.139 \times BMI + 0.718 \times \mathrm{log }\left(\gamma - GTP\right) + 0.053 \times WC - 15.745})}\right\}\times 100$$

### Statistical analysis

For descriptive statistics of the baseline characteristics, the median (IQR) was calculated for continuous variables, and frequency and percentage were calculated for categorical variables for the normal and RHF groups.

Univariate and multivariate analyses were performed by sex to assess the correlation between background and RHF. Multivariate adjusted logistic regression models were subsequently applied to evaluate the correlation of RHF with BMI categories (< 18.5, 18.5 ≥ to < 20, 20 ≥ to < 23, 23 ≥ to < 25, 25 ≥ to < 30, and ≥ 30) and FLI (10 equal parts), respectively. Multivariate analyses with three models were performed to calculate the odds ratios (ORs) and 95% confidence intervals (CIs). The models were adjusted for age (categorised into four age groups: 40–44, 45–49, 50–54 and 55–59 years) for Model-1; Model-1 plus FPG level (normal; FPG < 100 mg/dl, prediabetes 1; FPG 100–109 mg/dl, prediabetes 2; FPG 110–125 mg/dl), blood pressure level (Normal; SBP < 120 mmHg and DBP < 80 mmHg, prehypertension; SBP 120–139 mmHg or DBP 80–89 mmHg, hypertension; SBP ≥ 140 mmHg or DBP ≥ 90 mmHg), HDL, antihypertensive medication use, lipid-lowering medication use, current smoking, and daily drinking for Model-2; Model-2 plus, exercising for 30 min or more per day, skipping breakfast, midnight eating, weight gain of 10 kg or more since 20 years of age, and adequate sleeping for Model-3.

We also performed a sensitivity analysis using the Modification of Diet in Renal Disease equation (adapted for Japanese individuals by the Japanese Society of Nephrology) [25], as follows:

Estimated GFR (eGFR) (mL/min/1.73 m^2^) = 194 × serum Cr ^−1.094^ (mg/dL) x age ^−0.287^ (years) (× 0.739 if female).

In addition, a stratified analysis was performed, dividing the subjects into two groups: normoglycemia and prediabetes. The definition of prediabetes is HbA1c ≥ 5.7% or FPG ≥ 100 mg/dl, according to American Diabetes Association (ADA) criteria [26].

To evaluate the fitness of the model, we performed a lack-of-fit test [27].

All the tests were two-tailed, and the significance level was set to 0.05. For statistical analysis, JMP® version 15.0 (SAS Institute Inc., Cary, NC, USA) was used. The results are reported in accordance with the recommendations of the Strengthening the Reporting of Observational Studies in Epidemiology (STROBE) checklist [28].

## Results

### Study population

Of the 215,063 beneficiaries, data of 62,379 eligible subjects were extracted from the database.

### Baseline characteristics

Descriptive analyses for the baseline characteristics of the eligible RHF and normal filtration subjects by BMI class (lower 18.5, 18.5– < 21, 21– < 23, 23– < 25, 25– < 30, and 30 or higher) are shown in Table 1 for females and males, respectively. Every variable differed significantly among the groups.

**Table 1:**
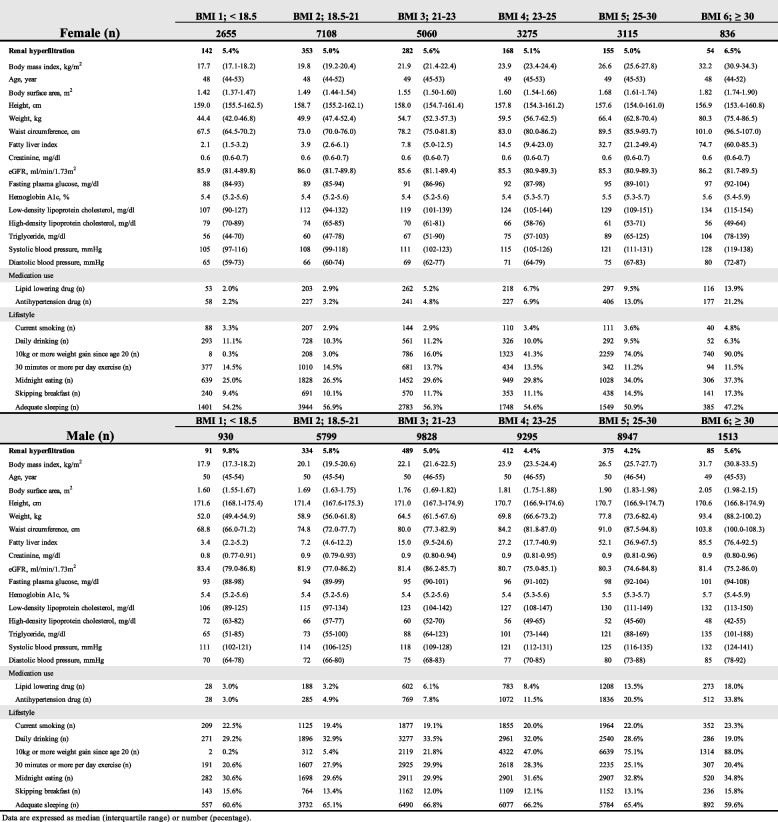
Characteristics of eligible subjects, excluding hypofiltration by body mass index in females and males, respectively

Descriptive analyses for the baseline characteristics of the eligible RHF and normal filtration subjects by 10 equal parts of FLI are shown in Table 2 for females and males, respectively.

**Table 2:**
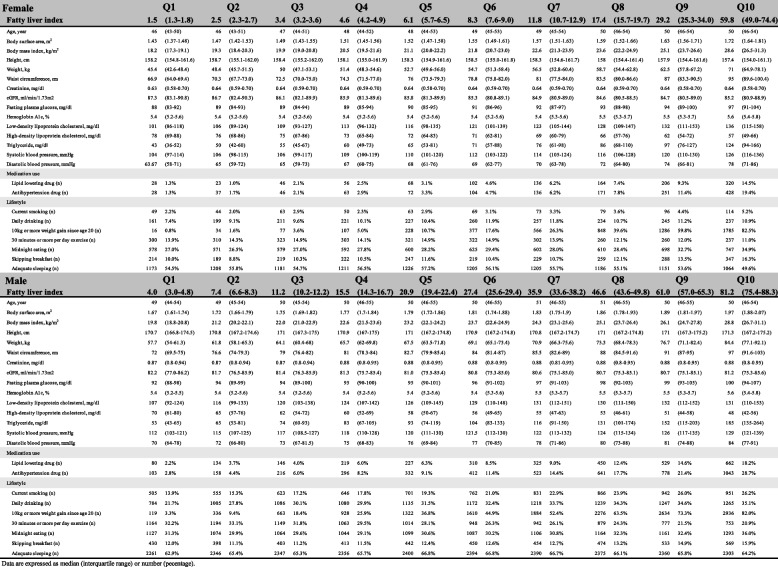
Characteristics of eligible subjects, excluding hypofiltration by 10 equal parts of fatty liver index in women and men, respectively

### Association of RHF and BMI or FLI level

The results of the multivariate adjusted logistic regression analysis for the three models are presented in Table 3. In women, negative correlation was noted for BMI < 21, and positive correlation was noted for BMI ≥ 30 for RHF in all the three models.

**Table 3:**
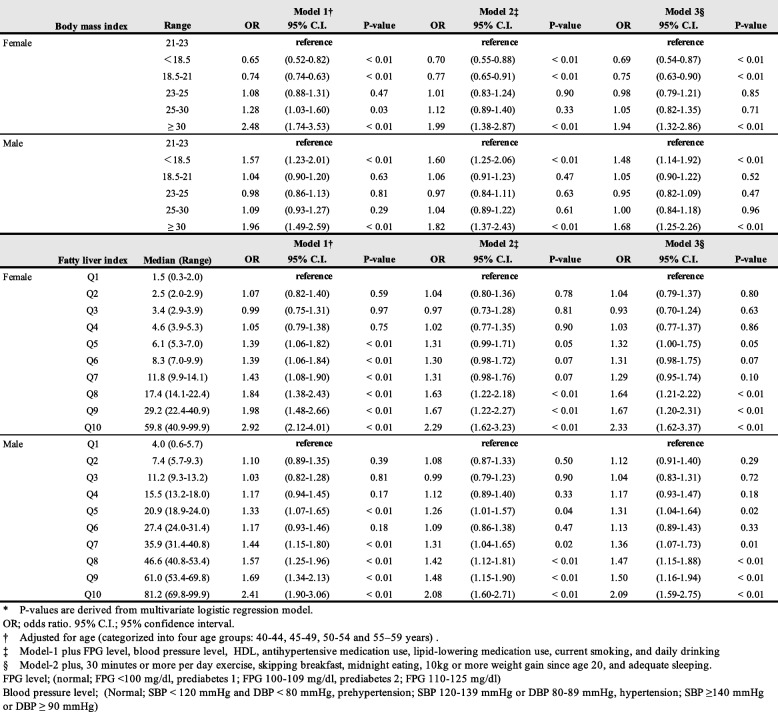
Multivariate adjusted logistic regression models for the prevalence of RHF by body mass index level or fatty liver index level, respectively. *　*P*-values were derived using the multivariate logistic regression model

FPG level: (normal FPG < 100 mg/dl, prediabetes 1; FPG 100–109 mg/dl, prediabetes 2; FPG 110–125 mg/dl)

Blood pressure level (normal, SBP < 120 mmHg and DBP < 80 mmHg, prehypertension; SBP 120–139 mmHg or DBP 80–89 mmHg, hypertension; SBP ≥ 140 mmHg or DBP ≥ 90 mmHg)

*OR* Odds ratio

^†^Adjusted for age (categorised into four age groups: 40–44, 45–49, 50–54, and 55–59 years)

^‡^Model-1 plus FPG level, blood pressure level, HDL, antihypertensive medication use, lipid-lowering medication use, current smoking, and daily drinking

^§^Model-2 plus, exercising for 30 min or more per day, skipping breakfast, midnight eating, 10 kg or more weight gain since age of 20 years and adequate sleeping

On the other hand, in men, BMI < 18.5 and BMI ≥ 30 were positively correlated with RHF in all three models.

In all the three models, there was a positive correlation of RHF with FLI values above 14.1 in women and above 35.9 in men.

**Fig. 3:**
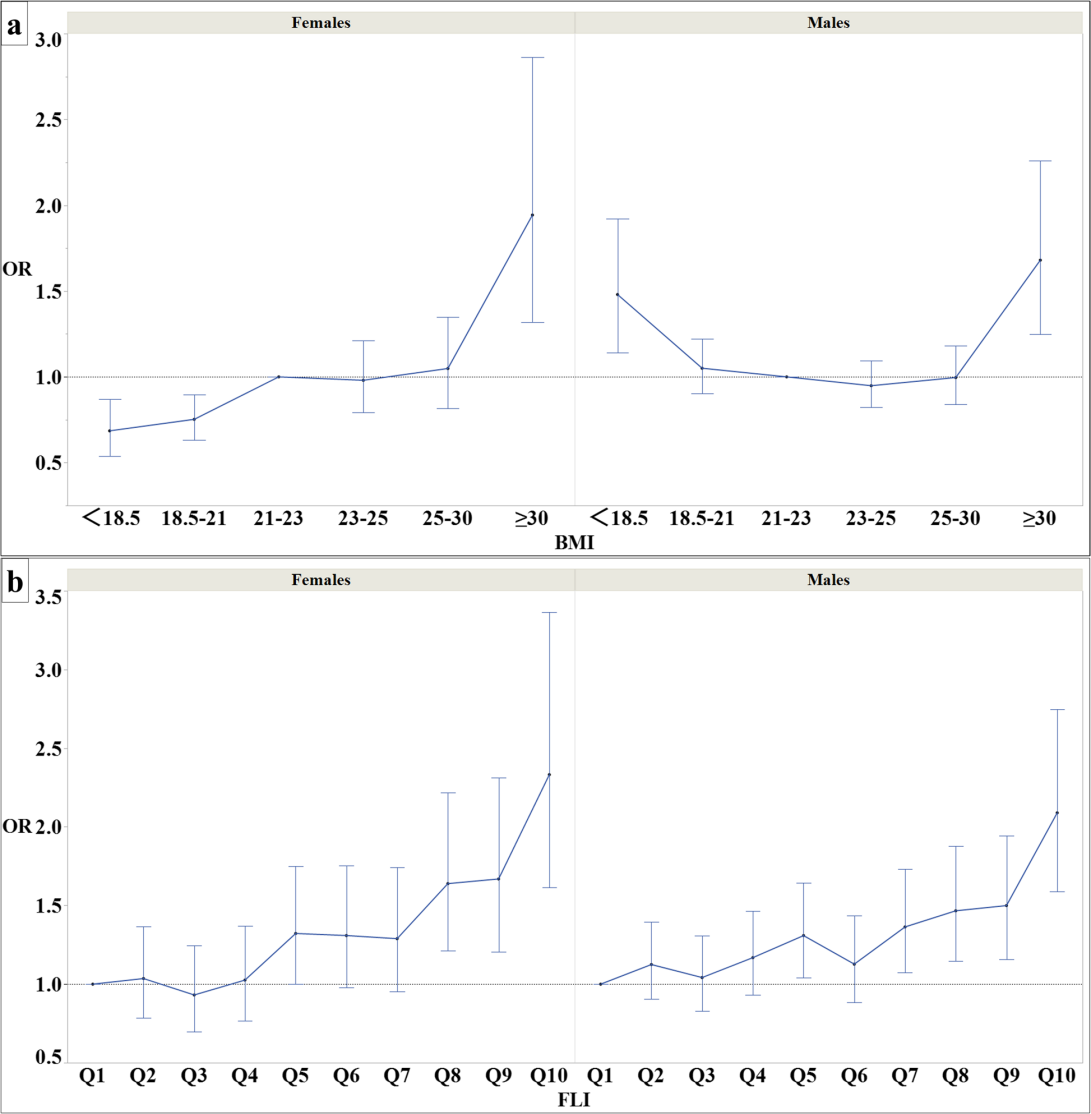
Graph of correlation between BMI and FLI for RHF. The dots represent each odds ratio for the categories classified by BMI values; additionally, the error bars represent 95% CI of the odds ratio

The results of the multivariate adjusted logistic regression analysis for BMI or FLI in Model 3§ are shown in Fig. 3.

**Fig. 4:**
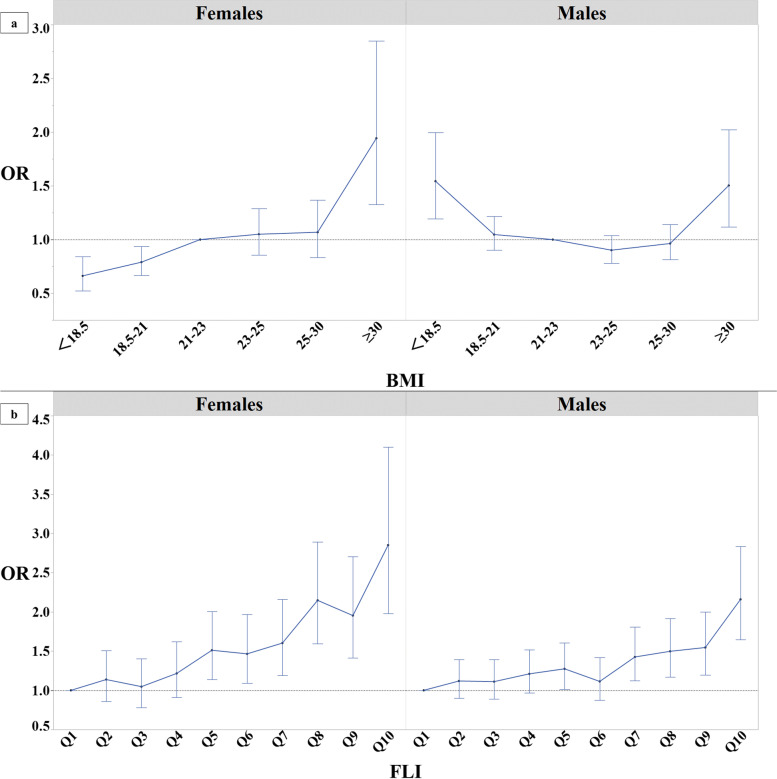
Graph of correlation between BMI and FLI for RHF (a sensitivity analysis). The dots represent each odds ratio for the categories classified by BMI values; additionally, the error bars represent 95% CI of the odds ratio

The results of sensitivity analysis using the Modification of Diet in Renal Disease equation (adapted for Japanese individuals by the Japanese Society of Nephrology), are shown in Fig. 4. There were no remarkable differences compared to using the CKD-EPI equation.

### Association of RHF and BMI by glycemia level

**Fig. 5:**
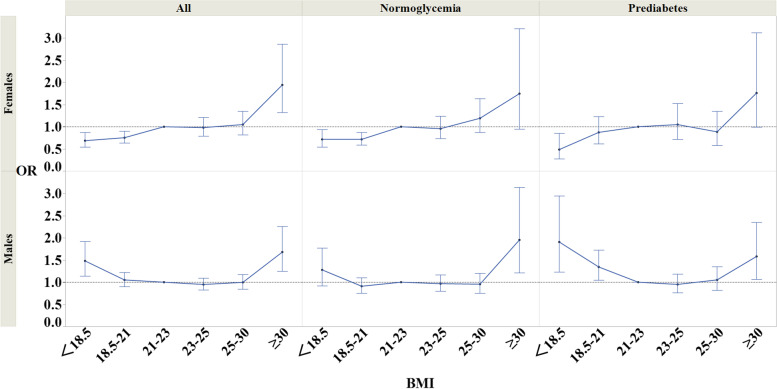
Graph of correlation between BMI for RHF (a stratified analysis, dividing the subjects into two groups: normoglycemia and prediabetes.). The dots represent each odds ratio for the categories classified by BMI values; additionally, the error bars represent 95% CI of the odds ratio

In this study, 58,361 of 62,379 non-diabetic subjects were analyzed, excluding hypofiltration. Of these, 22,657 (38.8%) were prediabetes. By gender, 15,823 (43.6%) of the 36,312 men and 6834 (31.0%) of the 22,049 women were prediabetes. The results of the multivariate adjusted logistic regression analysis for BMI in Model 3§ for the normoglycemia and prediabetes groups are shown in Fig. 5, respectively. In males, the OR for BMI < 18.5 was significant in the prediabetes group, but not in the normoglycemia group. In females, stratified analysis showed that the OR for BMI ≥ 30 was no longer significant in normoglycemia nor in prediabetes.

## Cut-off values of FLI for RHF by sex

The cut-off values for FLI in RHF were 14.7 and 30.4 for women and men, respectively.

## Discussion

In this study, we examined the correlation between BMI or FLI with RHF, respectively. For BMI, there was a positive correlation with RHF in women and a U-shaped correlation in men. On the other hand, there was a linear positive correlation with RHF in both men and women.

The association between BMI and RHF differed according to sex. In this analysis, BMI ≥ 30 was correlated with RHF in both sexes, which supports previous reports that obesity is a risk factor for RHF [29]. One result not seen in previous reports was that among men, the lean body mass index (BMI) < 18.5 was also associated with RHF. Although the relationship between low body mass and RHF has not been fully explained, it is widely known that the relationship between BMI and all-cause mortality is U-shaped, with the lowest rates between 22.5 and 25 kg/m^2^ [30]. Thus, low or high values of BMI are associated with an increased risk of mortality. Furthermore, several previous studies have argued that RHF is associated with mortality risk [7, 24]. Therefore, the relationship between low body mass and RHF may reflect a completely different pathology than the relationship between obesity and RHF.

It has been found that even in the absence of diabetes, high insulin resistance is likely to increase the renal intraglomerular hydrostatic pressure [31]. It is known that Asians, particularly East Asians, have a lower capacity for fat storage in subcutaneous adipose tissue, compared with other ethnic groups [32]. Therefore, lipid spillover, in which free fatty acid (FFA) overflow from adipose tissue, is thought to be more likely. Lipid spillover may result in the accumulation of ectopic fat, such as fatty liver, which may lead to insulin resistance. Kadowaki et al. evaluated the fat distribution, adipose tissue insulin resistance, and skeletal muscle insulin resistance in non-obese Japanese men [33]. Even among non-obese individuals, visceral and hepatic fat accumulations were observed in some individuals, with various accumulation patterns. Even in the absence of visceral fat accumulation, muscle insulin resistance (metabolic disturbance) was observed in the presence of fatty liver, whereas no insulin resistance was observed in the absence of fatty liver, even in the presence of visceral fat accumulation. It is notable that extremely thin people have lower muscle mass and are more likely to develop insulin resistance, which may lead to the development of RHF.

In terms of the relationship between FLI and RHF, it was linearly significant from Q8 for women (FLI > 14.1) and Q7 for men (FLI > 31.4). In addition, the cut-off values for FLI in RHF were 14.7 for women and 30.4 for men. The cut-off value of FLI for NAFLD in Asians is about 30; specifically, it is 35 for men and 20 for women [34, 35], which is generally consistent with the present results. It can be mentioned that FLI correlates well with RHF, and NAFLD and RHF might be associated. The FLI might be more useful than BMI in screening for RHF. However, cross-sectional epidemiological studies are not suitable for estimating the pathophysiology or causality. Therefore, further longitudinal studies and interventional trials are needed to further investigate the U-shaped association between RHF and BMI.

This study had several limitations. Because this study was cross-sectional, it was not possible to assume a causal relationship with RHF. There might have been some bias towards the participants who were particularly motivated to undergo a health check-up. Since most of the health check-up recipients are workers, a sampling bias due to the healthy worker effect is possible. Information on serum uric acid levels and uric-acid-lowering drugs was not available for this study.

## Conclusions

The BMI and RHF correlated linearly in women, but the correlation was U-shaped in men. On the other hand, FLI and RHF correlated linearly in both sexes. NAFLD may be associated with RHF. FLI is a simple marker that can be obtained from health check-ups. Since a high FLI correlated with RHF, it may be beneficial to monitor the renal function in such a population.

## Data Availability

Data cannot be shared publicly because of agreement between data holders. Data are available from The Mutal Aid Association of Prefectural Govermment Personnel (contact via https://www.chikyosai.or.jp/) for researchers who meet the criteria for access to confidential data.

## Abbreviation

RHF: Renal hyperfiltration
BMI: Body mass index
FLI: Fatty liver index
NAFLD: Non-alcoholic fatty liver disease
BSA: Body surface area
GFR: Glomerular filtration rate
CKD: Chronic kidney disease
MDRD: Modification of Diet in Renal Disease Study
CKD-EPI: Chronic Kidney Disease Epidemiology Collaboration
SBP: Systolic blood pressure
DBP: Diastolic blood pressure
HDL-c: High-density lipoprotein cholesterol
LDL-c: Low-density lipoprotein cholesterol
TG: Triglyceride
GGT: Gamma-glutamyl transferase
WC: Waist circumference
FPG: Fasting plasma glucose
Cr: Creatinine
